# Supplementary material for: Mutation-Based Antibiotic Resistance Mechanism in Methicillin-Resistant Staphylococcus aureus Clinical Isolates
Source: Pharmaceuticals (Basel). 2021 May 1;14(5):420. doi: 10.3390/ph14050420 (PMC8147353; doi:10.3390/ph14050420)
Supplement: Supplementary file 1 [file pharmaceuticals-14-00420-s001.zip › pharmaceuticals-1191809-supplementary.pdf]

Supplementary Data

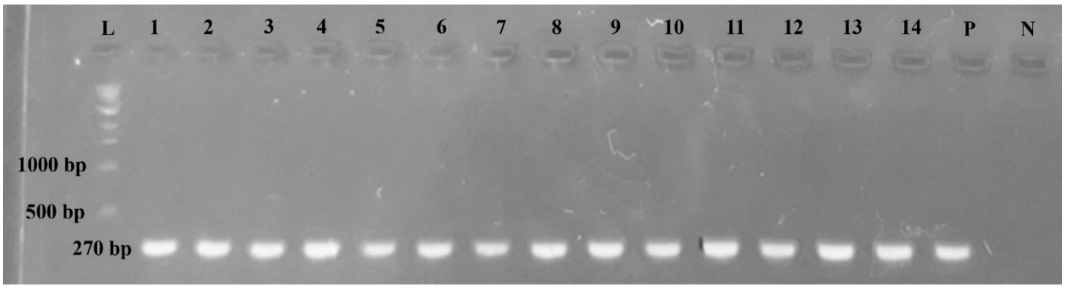

Figure S1. Amplification of *nuc* gene fragment (270 bp) through PCR from *Staphylococcus aureus* isolated phenotypically from clinical samples**.** Lanes 1-14 indicate representative *S. aureus* isolates (MR-1, MR-2, MR-3, MR-4, MR-5, MR-6, MR-7, MR-8, MR-9, MR-10, MR-13, MR-14, MR-15, and MR-33). Lane P, positive control; N, negative control; L, DNA marker.

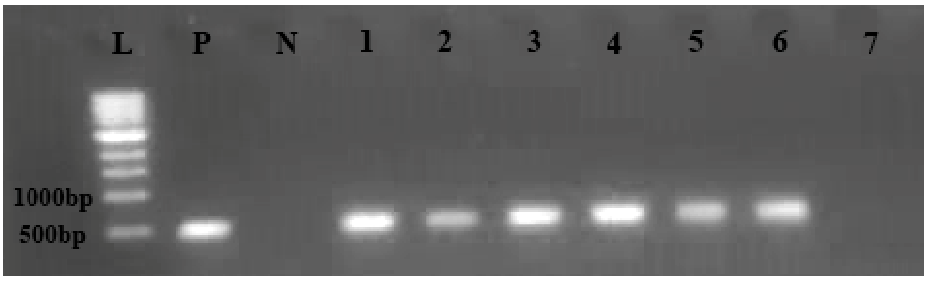

Figure S2. Amplification of *mecA* gene fragment (533 bp) through PCR from MRSA isolates**.** Lanes 1-6 indicate representative MRSA isolates (MR-1, MR-2, MR-5, MR-13, MR-14, and MR-33). MR-15 (lane 7) is *mecA*-negative. Lane P, positive control; N, negative control; L, DNA marker.

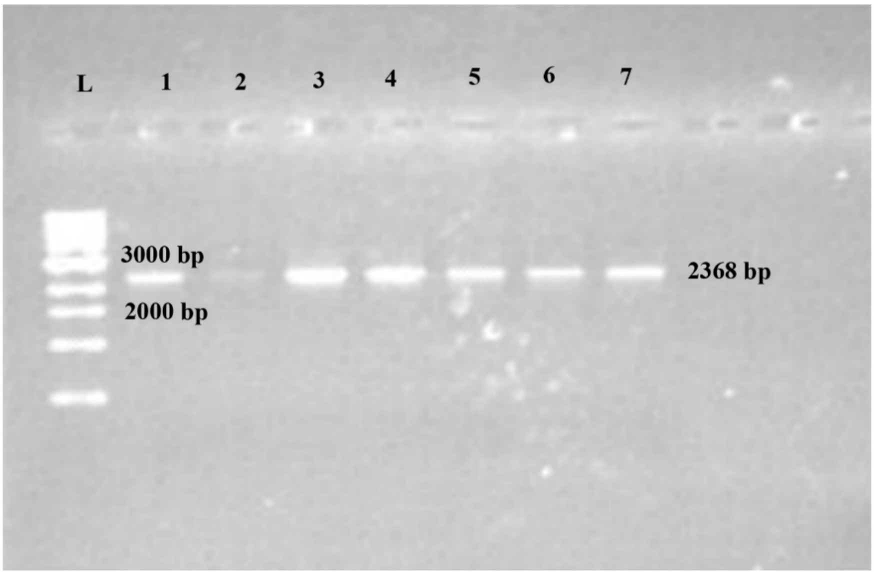

Figure S3. Amplification of the entire *mecA* gene region (2368 bp) through PCR from MRSA isolates**.** Lanes 1-7 indicate representative MRSA isolates (MR-1, MR-2, MR-5, MR-10, MR-13, MR-14, and MR-33). Lane P, positive control; N, negative control; L, DNA marker.
